# Pilot study on patients with Mal de Debarquement syndrome during pregnancy

**DOI:** 10.4155/fsoa-2018-0109

**Published:** 2019-02-21

**Authors:** Viviana Mucci, Josephine M Canceri, Yves Jacquemyn, Angelique Van Ombergen, Leen K Maes, Paul H Van de Heyning, Cherylea J Browne

**Affiliations:** 1 Translational Neurosciences, Faculty of Medicine & Health Sciences, University of Antwerp, Antwerp University Hospital, Wilrijkstraat 10 (Route 71–125), 2650 Edegem, Antwerp, Belgium.; 2 Department of Otorhinolaryngology & Head & Neck Surgery, Antwerp University Hospital, Wilrijkstraat 10 (Route 71–125), 2650 Edegem, Antwerp, Belgium.; 3 Swiss Concussion Center, Schulthess Klinik, Lengghalde 2, CH-8008 Zürich, Switzerland.; 4 Department of Neurology, Zurich University, University Hospital Zurich, Frauenklinkstrasse 26, 8091, Zürich, Switzerland.; 5 School of Science & Health, Western Sydney University, Room 21.1.12, Campbelltown Campus, NSW 2560, Australia.; 6 Department of Gynaecology, Antwerp University Hospital, University of Antwerp, Wilrijkstraat 10 (route 71–125), 2650 Edegem, Antwerp, Belgium.; 7 Ghent University, Department of Audiology, Faculty of Medicine & Health Science, University Hospital Ghent, Ghent University, Ghent, Belgium.; 8 Translational Neuroscience Facility, School of Medical Sciences, Room 316, Level 3, Wallace Wurth Building, UNSW Sydney, NSW 2052, Australia

**Keywords:** estrogen, Mal de Debarquement syndrome (MdDS), MdDS symptoms, pregnancy

## Abstract

**Aim:**

To evaluate if patients with Mal de Debarquement syndrome (MdDS) demonstrate different symptom levels or symptom type during pregnancy.

**Materials & methods:**

18 MdDS patients that were or had been pregnant during their condition were recruited to complete a retrospective online questionnaire. Respondents answered questions regarding their basic clinical data, diagnosis, triggers and differences in symptom level and symptom type during pregnancy and before pregnancy.

**Results:**

A total of 81.3% reported that their symptoms were reduced during pregnancy compared with before pregnancy. Respondents also reported a different perception of motion and experienced less dizziness while being pregnant.

**Conclusion:**

The physiological changes that occur during pregnancy improve the symptoms of patients with MdDS, and this is potentially attributable to the rise in estrogen and progesterone.

## Lay abstract

Mal de Debarquement syndrome (MdDS) is a rare neurological disorder characterized by a constant sensation of self-motion. More women are affected than men, and subsequently a hormonal implication has been theorized. This study aimed to evaluate if symptoms change in patients with MdDS during their pregnancy. A total of 18 MdDS patients were recruited to complete a retrospective online questionnaire. Among these, 81.3% of respondents reported that their symptoms were lower during pregnancy compared with before pregnancy. Respondents also reported a different perception of motion and experienced less dizziness while being pregnant. Our results support the hypothesis that pregnancy positively influences MdDS symptoms.

## Background

Changes in hormone levels, specifically estrogen and progesterone, influence numerous physiological systems [1], including the vestibular system, which affects inner ear functions [2]. In females, previous studies have shown that changes in the metabolism of estrogen and progesterone can lead to symptoms such as dizziness, tinnitus or sudden hearing loss, and can lead to peripheral and central vestibular alterations, especially during different hormonal stages (e.g., menses, pregnancy, menopause, etc.) [2–4]. Conversely, when considering the hormonal changes that occur during pregnancy exclusively, it has been shown that this hormonal milieu can improve symptoms in patients affected by migraine [5,6], as well as clinical pain conditions (e.g., arthritis) [7].

Mal de Debarquement syndrome (MdDS), which is French for ‘sickness from disembarkation’, is considered a neurological disorder characterized by a constant sensation of self-motion. When disembarking from a vehicle, subjects can often describe temporary sensations of unsteadiness and difficulty readjusting to a stable environment [8,9], termed ‘Mal de Debarquement’. In most cases, these symptoms resolve within days; however, when symptoms persist for months, and up to years, the condition is diagnosed as MdDS [8]. Currently, two onset subtypes have been identified, although a formal classification has yet to be established. The classic form of MdDS is termed motion-triggered (MT)-MdDS [10], as it typically starts following exposure to passive motion (e.g., cruises, car rides, flights, etc.). The second subtype is named spontaneous/other (SO) or Non-Motion-Triggered MdDS [11], where the same symptom profile arises spontaneously or following other non-motion events such as surgeries, childbirth, periods of stress, and so forth. [10]. MdDS is a debilitating condition, strongly impacting patient quality of life [12]. Previous research has identified that the distinguishing feature of MdDS is a persistent sensation of self-motion, which is typically described as rocking, swaying or bobbing [13], and is temporarily alleviated by being re-exposed to passive motion (e.g., driving in a car) [10]. This temporal relief of symptoms during passive motion clearly differentiates MdDS from other vestibular conditions. In addition to this, MdDS patients report associated symptoms, such as migraine [14], brain fog, unsteadiness, cognitive impairment, visual sensitivity, secondary mood disorders [15] and otological symptoms such as tinnitus and/or fullness of the ears [8]. While MdDS is considered an uncommon disorder [11], the actual prevalence of the condition is still unclear; however, its significant female predominance is well documented [8,10,15,16]. It is this high female predominance that has led to hypotheses that female hormones may be involved in the pathophysiology of MdDS [17,18]. More specifically, it has been hypothesized that hormonal fluctuations may be responsible for influencing MdDS onset and symptom fluctuations [14,19]. Although some anecdotal reports of changes in MdDS symptomatology are present, there are no published data about the effect of pregnancy on patients with MdDS. This could be due to the epidemiological characteristics of MdDS patients, as the average age of onset is in most cases occurring in the late 40s [10,20]. Consequently, the majority of female patients report experiencing perimenopause or undergoing hormonal replacement therapy during the occurrence of their MdDS onset [17]. Therefore, reproductively active females, who experienced pregnancy while suffering from MdDS, are the minority within this group. Nevertheless, given the hypothesis that ovarian hormones may influence symptom fluctuations, we believe that assessing patients with MdDS that are pregnant or that have been pregnant while having MdDS may develop our understanding of the underlying mechanisms of the condition and in turn aid in the development of patient management.

With regards to the pathophysiological mechanisms underlying MdDS, two main theories have been developed [21]. In the first theory, MdDS is hypothesized to be the result of a maladaptation of vestibular ocular reflex (VOR) [13]. The VOR is subjected to adaptation depending on the context [22–24]; for example, a cross-axis adaptation is present when traveling on a cruise ship, and a similar cross-axis re-adaptation will occur on returning to a static environment (i.e., land). However, in MdDS patients this mechanism seems to fail [21]. This is further reviewed in [13]. According to the second theory, MdDS is believed to be a disorder of abnormal functional connectivity. This theory was developed following findings from neuroimaging and neuromodulation studies on MdDS patients. According to these findings, MdDS can be considered as a disorder of oversynchronization of brain networks. This has been confirmed with MdDS patients responding favorably to neuromodulation [25], with the entorhinal cortex and amygdala as key neuronal areas [11]. For more information, see Cha [11].

Despite the formulation of these primary hypotheses to elucidate the underlying pathophysiology of MdDS, a unifying and clear theory has not been established. Similarly, it is still unclear why MdDS patients report symptom fluctuations [26], specifically, why female MdDS patients report an aggravation of symptoms during menses and ovulation [18,26]. A new hypothesis has been recently formulated, where particular hormonal phases are suggested to play a role in influencing neurotransmitters that are potentially involved in MdDS pathophysiology [19].

Another important characteristic of this patient group to consider is the high prevalence of migraine [27]. Symptom fluctuations in relation to female hormones have been previously described in Ménière's disease [28] as well as in patients affected by migraine [6]. Thus, the influence of ovarian hormones on MdDS symptomatology could be similar to the ‘estrogen-withdrawal theory’ that is well established in the pathophysiology of migraine in females [6,29]. Similarly, when considering migrainous patients, between 55 and 90% report to have an improvement in symptoms during pregnancy, regardless of the type of migraine they suffered from [30]. Hormones are able to alter neurological structure and functionality within the brain, and such changes have varied effects throughout the reproductive lifespan of a woman [31–34]. These observations may be relevant when considering MdDS as a disorder of neuroplasticity, where ovarian hormones may influence or modulate brain functionality which consequently leads to symptom changes.

Pregnancy is known to induce physiological and biochemical changes within the body [35], for example, the monthly cyclical fluctuations of ovarian hormones cease and an increase in estrogen and progesterone levels is observed. The changes in ovarian hormone levels are also known to affect pain perception [7]. If considering migraine (namely menstrual migraine), 11% of patients report an improvement of symptoms within the first trimester, which rises to 53% in the second and 79% in the third trimester [36]. During the postpartum phase, estrogen levels rapidly fall leading to an exacerbation of symptoms with 34% of women suffering a relapse of migraine symptoms, reaching up to 55% within a month after delivery [36]. The improvement reported in migraine, as in other clinical pain conditions, is termed pregnancy-induced analgesia [37], where hormonal changes as well as changes in neurotransmitters [19], including GABA, serotonin, and endorphins, are believed to be responsible for these improvements. Currently, it is unclear how ovarian hormone fluctuations during pregnancy may or may not influence female MdDS patients. As a result, this study aimed to conduct a preliminary pilot questionnaire, in order to evaluate if MdDS patients report differences in symptom levels or symptom type during their pregnancy. This study also aimed to examine how many women developed MdDS while pregnant and if hormonal changes were involved in triggering the onset itself. Additionally, this study sought to compare the two onset groups (SO and MT) and evaluate potential differences where possible. We hypothesized that MdDS patients would report a similar pattern of symptom improvement to migrainous patients during pregnancy. This study may provide us with valuable information about the effects of ovarian hormones on MdDS symptomatology and lead to the improvement of patient care during pregnancy.

## Methods

### Ethical approval/study population & recruitment

Ethical approval was provided by the Ethics Committee of the University Hospital Antwerp Belgium (IRB number 15/44/454) and by the Western Sydney University Human Ethics Committee (H11962). Each respondent gave informed consent. All investigations were performed according to the principles expressed in the Declaration of Helsinki.

Patients diagnosed by specialists or believing to suffer from MdDS (also referred to as self-diagnosed patients) were included in the study. Patients were recruited across the USA, Europe and Australia. MdDS patients were recruited through the Department of Otorhinolaryngology at the University Hospital of Antwerp, Belgium. Patients were also recruited globally through the main MdDS support groups: MdDS Australia Facebook Support Group, MdDS UK Facebook Support Group, website of MdDS Research Group at Mount Sinai Hospital, Western Sydney University MdDS Research Group Facebook page, website and Facebook of Vestibular Disorders Association and website and Facebook of Whirled Foundation and the REACT Community Facebook.

### Inclusion & exclusion criteria

The Inclusion criteria used for this study based on the most recent diagnostic guidelines [10]. Female patients (aged above 18 years old) who were pregnant while suffering from MdDS were considered. In order to be diagnosed with MdDS, patients had to report persistent Mal de Debarquement symptoms (rocking, swaying, bobbing) for more than 1 month. Patients with an MT or SO onset were included. Self-diagnosed patients were included, but only if they fit within the recent guidelines [10]. Patients reporting symptoms that did not fit the guidelines were excluded. Male patients, and female MdDS patients who were not and never had been pregnant while suffering from MdDS, were excluded from this study.

### Questionnaire

The questionnaire was distributed online using the survey platform Qualtrics^®^, comprising 45 questions (see Supplementary Material) which was divided into separate categories for each subtype (MT and SO): epidemiology (demographic details), diagnosis (i.e., who made the initial diagnosis), onset triggers, symptom triggers (i.e., symptom fluctuation, assessments of potential triggers), hormonal influences, symptom comparison (level and type) between pregnancy and when not pregnant, and symptom fluctuations (e.g., how symptoms varied during the 9 months of pregnancy and after). Respondents were also given the chance to add comments (open-ended comment section) regarding their symptoms, triggers or to provide any information they considered relevant.

### Statistical analysis

Statistical analysis was performed with SPSS version 24 (IBM Corp). Nonparametric Chi Square was used for comparison between MT and SO groups. When the minimum expected count in Chi Square analysis was <5, the two-sided significance was then considered. If no statistical significant difference was observed between the two groups, the group data were merged analysed as one. Nonparametric Wilcoxon test (two-tailed) was used to evaluate the improvement in the rating of symptoms. Repeated measures analysis of variance (ANOVA) was used to analyse how symptoms changed according to triggers (e.g., different positions, bright lights, etc.) before as well as after pregnancy.

## Results

### Epidemiology & diagnosis

A total of 18 respondents were included in the study as per the described inclusion and exclusion criteria. Not all questions were relevant to all respondents, for example; respondents who had developed MdDS during pregnancy were not required to answer questions about their experience with MdDS prior to pregnancy; therefore, results are individually based upon the number of respondents who completed each question.

The majority of the respondents were from North America with 61.1% (n = 11), 38.9% of respondents were from Australia and Europe (n = 4 and n = 3, respectively). The average age was 38 years (SD = 5.1, ages ranged from 28 to 47 years). Four of the respondents were of the SO subtype and fourteen of the MT subtype. The 88.9% of the respondents (n = 16) had MdDS prior to becoming pregnant and two respondents (11.1%) reported that their MdDS symptoms started during pregnancy. Seven respondents who participated in the study had previously been pregnant while experiencing MdDS symptoms (previously pregnant), while, 11 respondents were currently pregnant at the time of completing the question (currently pregnant). Both groups were treated in the same way. The majority of the currently pregnant women were between week 13 and 25 of gestation when they completed the questionnaire. When considering the diagnostic experience of respondents, the majority were diagnosed by neurologists – 38.9% (six MT and one SO), followed by otolaryngologists – 27.8% (three MT and two SO). Three respondents (two MT and one SO) were self-diagnosed (16.7%), 11.1% (two MT) were diagnosed by physiotherapists and one MT respondent (5.6%) was diagnosed by a general practitioner.

### Onsets

As reported in Table 1 of the 18 responses collected, the majority of the respondents had an MT onset from a cruise or from some sort of water travel (55.6%), followed by flights, a combination of vehicles and anxiety/panic attacks (each 11.1%).

**Table 1.** **Onset triggers reported by respondents within the motion-triggered and spontaneous/other groups expressed as the percentage of respondents and number of respondents (n).**

**Onset trigger**	**Motion-triggered (n = 14)**	**Spontaneous/other (n = 4)**
Cruise	55.6% (n = 10)	

Flight	11.1% (n = 2)	

Combination of vehicles	11.1% (n = 2)	

Anxiety/panic attack		11.1% (n = 2)

Pregnancy		5.6% (n = 1)

Unknown		5.6% (n = 1)

Respondents were asked about how long they had suffered with MdDS symptoms prior to getting pregnant, to which the majority (38.9%; 27.8% MT and 11.1% SO) reported that they had experienced MdDS for 3–4 years prior to falling pregnant (Table 2).

**Table 2.** **Duration of Mal de Debarquement syndrome before pregnancy reported by respondents within the motion-triggered and spontaneous/other groups expressed as the number of respondents (n) and percentage of respondents for both groups.**

	**Motion-triggered**	**Spontaneous/other**
0 year (MdDS onset during pregnancy)	1 (5.6%)	1 (5.6%)

<6 months	1 (5.6%)	0 (0.0%)

1–2 years	3 (16.7%)	1 (5.6%)

3–4 years	5 (27.8%)	2 (11.1%)

5–6 years	2 (11.1%)	0 (0.0%)

7–10 years	1 (5.6%)	0 (0.0%)

### Hormonal contraceptive use before pregnancy

Respondents were asked: *“Were you on any form of hormonal contraceptive prior to being pregnant?”*, 44.4% (n = 8) answered positively to this question (of these respondents – 75% had used oral hormonal contraceptives, 12.5% used hormonal implants and 12.5% a hormonal patch). 55.6% (n = 10) did not use hormonal contraceptives prior to pregnancy. The respondents that had used a hormonal contraceptive prior to pregnancy had been using them for, on average, 6.5 years (SD 4.9 years). The two onset groups were not distinguished.

### Symptom changes throughout pregnancy

Respondents were asked about their symptoms during pregnancy: “*What are/were your symptoms like during pregnancy compared with before pregnancy*”. This question was only presented to the respondents that had MdDS prior to pregnancy (n = 16). 13 respondents (81.3%) indicated that their symptoms improved during pregnancy, specifically 31.3% felt slightly better and 50.0% felt significantly better. Only one respondent (6.3%) reported no changes in their symptoms, and two respondents indicated that their symptoms were worse; specifically, one respondent felt slightly worse (6.3%) and another felt significantly worse (6.3%). No distinction between MT and SO was performed due to a small number of SO respondents. Respondents who indicated that their symptoms improved during pregnancy (n = 13) were given a follow-up question to identify which stage of pregnancy the improvements were experienced during (Table 3).

**Table 3.** **The pregnancy stages where symptom improvements were reported, (n) the number of respondents and (%) the percentage of respondents are presented.**

	**n = 13**
Better in first trimester	1 (7.7%)

Better during the first and second trimesters	1 (7.7%)

Better during the second and third trimesters	1 (7.7%)

Better overall during pregnancy	7 (53.8%)

Remission of symptoms	3 (23.1%)

As described in Table 3, the majority of respondents that reported an improvement of symptoms during pregnancy (7 out of 13–53.8%) indicated that their symptoms were ‘better overall during pregnancy’ compared with before pregnancy, and three respondents (23.1%) reported a complete remission of symptoms.

All respondents were asked if and when they experienced dizziness according to their position during their pregnancy (i.e., when standing, sitting or lying down). The majority reported to have no dizziness during pregnancy (n = 10–55.6%), with no differences among onset groups. A total of 11.1% of respondents indicated experiencing dizziness while seated (one MT and one SO), 16.7% when standing and 16.7% when lying down.

Respondents were asked to describe the nature of their symptom pattern during pregnancy, 33.3% of respondents reported that their symptoms were stable throughout pregnancy (five MT and one SO), and 33.3% of respondents reported that their symptoms fluctuated day by day throughout pregnancy (four MT and two SO). A total of 16.7% of respondents reported that their symptoms appeared to fluctuate randomly (two MT and one SO), 11.1% of respondents reported that their symptoms fluctuated depending on stressors (one MT and one SO) and one MT respondent indicated that the symptoms were cyclical in nature.

### Symptom ratings on good & bad days

Respondents were asked to rate their overall symptom perception during a good day (low MdDS symptoms) and during a bad day (high MdDS symptoms*)* on the following scale: ‘0 = symptom-free/10 = severe symptoms’. Specifically, they were asked to rate their symptoms prior and during their pregnancy. When analyzing the data for symptoms on a good day (low MdDS symptoms), a statistical difference was observed (p < 0.001 – Wilcoxon test). A significant reduction in symptoms was reported when comparing the ratings of a good day during pregnancy (0.61 [SD = 1.19]), to the ratings of a good day prior to pregnancy (2.63 [SD = 1.82]). On the bad day (high MdDS symptoms) data, a statistical difference was also observed (p = 0.013 Wilcoxon test) when comparing the bad day symptom levels during pregnancy (3.83 [SD = 2.57]) to prior to being pregnant (6.44 [SD = 2.39]).

### Motion perception

**Figure 1.**
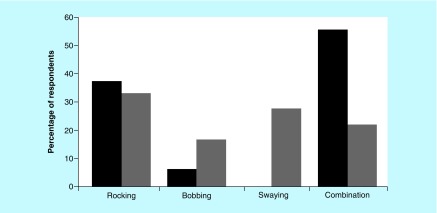
**Reported perception of motion by the respondents before and during pregnancy.** The number of respondents reporting more mixed/combined sensations of motion decreased during pregnancy, where the perception of motion seems to become clearer and solely involved one direction (e.g., only bobbing or swaying). Black bars: before pregnancy; Gray bars: during pregnancy.

Respondents were also asked which motion symptoms they experienced the most (i.e., bobbing, swaying, rocking or a combination) prior to and during pregnancy, and a significant difference was recorded (p < 0.001 – Mauchly's test), as reported in Figure 1.

Despite the small number of SO respondents, a statistically significant difference was observed between the two onset groups (p = 0.046 – before pregnancy; p = 0.016 – during pregnancy), where the SO group reported more rocking (forward and backward) than the MT overall during as well as prior to pregnancy. The MT group reported to have a greater sensation of a combination of directions prior to pregnancy, which changed to experiencing one predominant direction during pregnancy.

### Position & symptom aggravation

Respondents were also asked about which position affected their motion symptoms (e.g., standing, lying down, sitting) prior as well as during pregnancy. No significant differences were reported, indicating that different positions during pregnancy did not influence symptoms. However, while only two respondents (one MT, one SO) reported to have symptoms when lying down prior to being pregnant, during pregnancy the number of subjects reporting symptoms when lying down increased to seven (five MT, two SO).

### Triggers

In addition to this, the questionnaire inquired after MdDS symptom triggers. Symptoms were mainly aggravated by: a lack of sleep (10/18 respondents; 55.5%), stress (44.4%), bright lights (44.4% of respondents) and phone/computer use (38.8%; Table 4). Similarly, due to the limited number of SO respondents, the two onset groups were not separated and compared.

**Table 4.** **Various triggers that aggravated symptoms of respondents during pregnancy.**

**Triggers**	**The number of respondents (n/%) whose symptoms were aggravated**
After a car ride	6 (33.3%)

Drinking caffeinated beverages	3 (16.6%)

**Computer/phone use**	**7 (38.8%)**

In a department store	6 (33.3%)

In a supermarket or a grocery store	5 (27.7%)

Watching movies	3 (16.6%)

Weather changes	5 (27.7%)

**Stress**	**8 (44.4%)**

**Lack of sleep**	**10 (55.5%)**

Dehydration	2 (11.1%)

Hunger	5 (27.7%)

Food sensitivities	1 (0.05%)

Loud noises	2 (11.1%)

**Bright lights**	**8 (44.4%)**

Flashing lights	6 (33.3%)

Sources of vibration	4 (22.2%)

Elevator use	5 (27.7%)

Escalator use	5 (27.7%)

The total number (n) and percentage (%) of respondents that indicated that a certain trigger aggravated their symptoms is reported. Respondents were able to choose multiple answers if they had been triggered by any of the various events/situations (highlighted in bold the highest prevalent triggers).

### Mood

Respondents were asked: *“Does or did your mood influence your symptoms?”* The majority of respondents indicated that mood influenced their symptoms during pregnancy (55.5% [seven MT; three SO]).

### Symptom management

Respondents were asked if they had discussed their symptoms with a gynecologist, with the majority (77.7%) answering negatively to this question. A small number of respondents (4 MT) discussed their condition with their gynecologist and all four reported that their gynecologist was not aware of this syndrome, while the majority of respondents did not communicate about MdDS with their gynecologist.

### Open-ended comments

In total, 18 comments were left from the respondents. Eight comments explicitly reported that they felt better during pregnancy (examples reported: “symptoms went away during first two trimesters and came back in the last trimester”; “zero symptoms while being pregnant”; “overall I feel my symptoms are improving, but my symptoms were already improving prior to falling pregnant”; “overall my symptoms were much better during pregnancy, which I attributed to hormones”). Five respondents reported symptom aggravations due to stress, anxiety, weather changes and mostly visual triggers (e.g., scrolling/working on the computer, watching action TV, being in a supermarket).

## Discussion

This was the first pilot study made available to pregnant women suffering from MdDS. In order to collect a great number of respondents, an international multi-institutional collaboration was set up. This manuscript presents the data from a small number of subjects who met the inclusion criteria of being pregnant while suffering from MdDS. Therefore, the results presented should be considered as preliminary. From the results collected our hypothesis is confirmed, with a high number of MdDS sufferers reporting a change and improvement of symptoms during pregnancy.

### Epidemiology: diagnostic & onset

The majority of respondents were from North America; this could reflect the greater awareness in USA and Canada compared with Australia and Europe. With regard to diagnosis, specifically considering the healthcare professionals providing MdDS diagnoses, our results parallel a recently published study, where neurologists and otolaryngologists were the primary health professionals diagnosing MdDS patients [10]. Also, considering the onset triggers, the majority of the respondents were triggered by a motion event, namely after a cruise or sea travel, and this too is in line with the data recently published from our group [10]. Overall, in the current study, the number of SO respondents was limited, thus comparison of the two onsets (MT and SO) was impractical. With regards to onset duration, most of the MdDS respondents engaged in this pilot study had MdDS for on an average 3–4 years prior to becoming pregnant. Only two respondents reported that their MdDS symptoms began during pregnancy. Thus, given the small number of respondents, it is difficult to know if pregnancy and the associated hormonal changes exist as a trigger for MdDS symptoms. Respondents were queried about the usage of hormonal contraceptive prior to pregnancy. These data proved insignificant, preventing a correlation between hormonal contraceptive use prior to pregnancy and the influence on MdDS symptoms to be established.

### MdDS symptoms during pregnancy & underlying theories on the mechanism involved

The majority of MdDS respondents, regardless of subtype, reported an improvement of symptoms during pregnancy; however, they were unable to report accurately when the greatest improvement occurred. A high number of respondents reported an overall improvement throughout the whole pregnancy period, which was further supported by the open-ended comments. The second largest number of respondents reported to have felt better within the first two trimesters of pregnancy and only two respondents reported to have their symptoms aggravated throughout the pregnancy period. Most respondents also reported no dizziness during pregnancy. Hormonal changes occur immediately to conception; the trophoblast releases high levels of human chronic gonadotropin, allowing the corpus luteum within the ovary to continue to produce estrogen and progesterone until the formation of the placenta is complete [6]. Human chronic gonadotropin levels significantly reduce around week 16–20, and the levels of estrogen and progesterone steadily increased throughout pregnancy. Considering this, it is possible that the improvement of MdDS symptoms observed may be due to the absence of hormonal cyclic fluctuations and/or the high concentration of these hormones during pregnancy. This could support our hypothesis, that MdDS patients, similar to migraine patients [5], are subjected to symptom improvement during pregnancy via a hormonal mechanism. The placenta is responsible for producing the majority of estrogen and progesterone necessary for the progression of pregnancy [38]. The rise in estrogen and progesterone begins during the 6th to 8th week of pregnancy and continues to gradually increase to peak levels during the third trimester; serum estradiol levels during the third trimester are 30- to 40-times higher and progesterone levels are 20-times higher than their highest level during natural menstrual cycles [6,35]. It can therefore be suggested that the absolute level of these hormones experienced during pregnancy, contrasting normal cyclic fluctuations and the erratic changes seen during the menopausal transition, could be responsible for the symptom reduction demonstrated in this pilot study.

The higher level of estrogen and progesterone may also induce central nervous changes. Estrogen acts via two nuclear receptors, estrogen-receptor α and β [39]. These receptors operate as transcription factors via genomic mechanisms, regulated by altered expression of target genes [40], as well as by excitatory action within the central nervous system [41]. These receptors have been found in brainstem vestibular nuclei concerned with optokinetic, vestibular-ocular and vestibulo-spinal reflex [42]. Additionally, estrogens are known to facilitate the glutamatergic system, potentially enhancing neural excitability. Progesterone is able to activate GABAergic systems, suppressing neural activity [6]. A new recent hypothesis has been formulated considering the two pathophysiological theories previously described [13,19,21,25]. Within this hypothesis, hormonal fluctuations are considered to modulate the GABAergic system and thus influence key components involved in MdDS pathophysiology [13,19]. However, for now this remains just a theory.

### Nature of symptoms

With regard to the nature of symptoms, most respondents reported stable symptoms, followed by symptom changes day-to-day. This fluctuation could indicate that the symptom modulation is associated with the daily neuroendocrinological fluctuations, for example, the varying levels of cortisol. Cortisol is known to be associated with stress and is known to modulate throughout the day. Changes in the hypothalamo–pituitary–adrenal axis that also occur during pregnancy [33] may too influence MdDS symptoms. This hypothesis could be further evaluated. The necessity to understand the daily symptom modulation could lead not only to greater insights in MdDS pathophysiology, but also improvement of treatment options.

Additionally, the respondents were required to rate the severity of their symptoms on a scale of 0–10, during what they considered a ‘good day’ and a ‘bad day’, both prior to and during pregnancy. A statistically significant difference was reported when considering a good and a bad day prior to pregnancy and during the pregnancy period. Our results substantiate our hypothesis of an overall improvement of perception of symptoms during pregnancy. This was further supported by the open-ended comments, where respondents also reported an overall improvement in symptoms during pregnancy. Similarly, when comparing the respondents’ motion perception, most experienced a combination of motion directions (e.g., combination of swaying and bobbing) before pregnancy, which changed to experiencing mainly one direction (e.g., only mild swaying) during pregnancy, as reported in Figure 1. This could be the result of the overall reduction of symptoms. Despite a significant difference observed between self-motion perception in SO and MT subjects, the number of SO respondents in this study was too small to draw a firm conclusion. SO respondents reported a rocking sensation overall before and during pregnancy, something different from the MT group, where no rocking sensations were described before and during pregnancy. Further examinations about motion direction and phantom motion perception should be explored within the two onset groups, this could provide additional information about subtype differences.

Furthermore, respondents were asked if different positions (e.g., standing or lying down) affected the level of their symptoms. The majority of respondents reported experiencing a higher level of symptoms predominantly while lying down during pregnancy, something that was not common prior to being pregnant. These results could be due to supine hypotension syndrome (also referred to as aortocaval compression syndrome), which is caused when the gravid uterus compresses the aorta and inferior vena cava when a pregnant woman lays down in a supine position [43]. This leads to a decrease of venous return and a reduced blood flow. Within 3–10 min in the supine position, symptoms such as dizziness, pallor, low blood pressure, sweating and nausea and an increase in heart rate occur [43,44]. A total of 8% of pregnant women within the second and third trimesters of pregnancy may experience this [44]. In our research cohort, although it may be possible that MdDS respondents experienced supine hypotensive syndrome, we did not ask this specific question. As a result, it is difficult to determine why this particular position resulted in heightened symptoms during pregnancy. Generally, pregnant MdDS women should avoid conditions that can naturally increase the risk for triggering dizziness, such as lying down in the supine position, but should be advised to lie upon their left side.

### Triggers: mood

The respondents were also asked if during pregnancy they were more sensitive to a series of triggers known for triggering MdDS patients [20]. From the results collected, most respondents seemed to report a heightened sensitivity to visual stimuli (bright lights, phones, computers), which is a known phenomenon for MdDS patients [8]. It was also reported that mood and lack of sleep could influence symptoms, and stress was considered a trigger, coinciding with previous studies [10]. High levels of anxiety were also expressed in the open-ended comment section; again anxiety is considered a major trigger in influencing MdDS symptoms [10,20]. Overall respondents reported that their mood was able to influence their symptoms during pregnancy [45,46]. An aspect to consider with regard to mood is the increase of allopregnanolone, a neuroactive metabolite of progesterone [46]. Studies have shown that low levels of this hormone may be associated with depression, while higher levels, such as during pregnancy, may be able to improve mood [46]. Considering MdDS patients, the high levels of allopregnanolone during pregnancy may be involved in the overall reduction of symptoms.

### Symptom management

From our preliminary results, respondents reported that their gynaecological healthcare professionals were not aware or familiar with MdDS, which is understandable considering the condition is outside the scope of their medical speciality. However, given the drastic changes in MdDS symptoms potentially related to hormonal changes, it may be relevant to assess a patient's hormonal profile and to pursue follow-ups in the puerperium phase in collaboration with a gynecological consult. After delivery, ovarian hormonal levels drop and, as observed in migraine sufferers [5], MdDS patients’ symptoms may similarly return.

### Limitations

The survey questions requested information about events that may have occurred many years prior to pregnancy (MdDS onset), and no specific strategies for reducing recall bias were implemented. The number of MT and SO respondents was limited due to the difficulties in recruiting respondents, and a larger cohort should be considered, although we expect that patients with MdDS that have been pregnant are a clear minority within the larger MdDS patient population. Respondents were only queried as to how symptoms changed during pregnancy in respect to prior to being pregnant. A future assessment should consider follow-up on how symptoms may have changed postpartum.

## Conclusion

This was the first pilot study performed on pregnant MdDS patients and MdDS patients who had been pregnant while suffering from MdDS. This study indicates that most MdDS patients reported to have no dizziness during pregnancy, providing the foundation for further assessments.

Respondents, regardless of their onset type (MT or SO), reported overall an improvement of symptoms during pregnancy compared with before pregnancy, suggesting a potential beneficial influence of higher estrogen and progesterone concentrations. Subsequent studies should examine how symptoms may change in MdDS patients during each trimester as well as after delivery. These preliminary data may provide incentive for further investigation into the role of hormones in the symptom profile and pathophysiology of MdDS.

## Future perspective

As more research is conducted into MdDS, it is becoming clearer that hormones could play a significant role in the condition. To further understand MdDS symptom fluctuations and pathophysiology, more hormonal assessments and selected imaging studies should be performed. For example, imaging studies involving *in vivo* ^1^H-MRS images could allow the understanding of how MdDS subjects’ brains may respond to various hormonal states (e.g., during menstruation or ovulation). Future studies should aim to understand if hormonal changes are able to affect certain brain regions that have been implicated in MdDS pathophysiology.

**Summary points**

- Pregnant women suffering from Mal de Debarquement syndrome were queried about any symptom variations during pregnancy.
- A total of 81.3% of the respondents reported an improvement of symptoms during pregnancy (lower symptoms).
- Respondents reported to experiencing a differing perception of motion compared with before being pregnant.
- Improvement of symptoms may be attributed to the rise in estrogen and progesterone throughout pregnancy or to the absence of their monthly cyclic fluctuations.
- This study supports the theory of female hormones being involved in Mal de Debarquement syndrome pathophysiology.
- A longer follow-up, including delivery and after birth, should be considered for future studies.
