# Supplementary material for: Pilot study on patients with Mal de Debarquement syndrome during pregnancy
Source: Future Sci OA. 2019 Feb 21;5(4):FSO377. doi: 10.4155/fsoa-2018-0109 (PMC6511939; doi:10.4155/fsoa-2018-0109)
Supplement: Supplementary file 1 [file fsoa-05-377-s1.docx]

**Supplementary Material**

|   **1. BASIC INFORMATION** |
| --- |
| **1.1: Name:** |
| **1.2: Country/State/City:** |
| **1.3: Date of Birth:** |

| **2. PREGNANCY, DIAGNOSIS AND SYMPTOM INFORMATION** |  |
| --- | --- |
| **2.1: Are you currently pregnant or have you experienced MdDS symptoms while being pregnant?**  [ ]Yes  [ ] No |  |
| **2.2 Did you have MdDS prior to your pregnancy?**  [ ]Yes  [ ] No |  |
| **If YES to prior MdDS: 2.3a: Who initially diagnosed you with MdDS:**  *Select one answer:*    [ ] Neurologist  [ ] Otolaryngologist  [ ] Physiotherapist  [ ] ENT  [ ] Self-diagnosed  [ ] Other  **2.4a: Who diagnosed you with MdDS after your initial diagnosis: (e.g. if you were self-diagnosed initially and received an official diagnosis subsequently) and was the specialist/health care professional confident in the diagnosis?**  *Select one answer: (check box will appear next to options listed below for ‘confident in diagnosis’ response)*  [ ] Neurologist  [ ] Otolaryngologist  [ ] Physiotherapist  [ ] ENT  [ ] Other  [ ] N/A as initial diagnosis was the only diagnosis  [ ] N/A as self-diagnosed is the only diagnosis |  |
| **2.5a:**  **To the best of your knowledge, what was the event that induced your MdDS?**  [ ] A motion event (e.g. boat ride, flight, etc.)  [ ] No clear cause  [ ] Other (e.g. surgery, panic attack, etc.)  **2.6a: Please describe what you believe induced your MdDS**  **2.7a: How long did you have MdDS before becoming pregnant?**  [ ] 6 + months  [ ] 1 -2 years  [ ] 3-4 years  [ ] 5-6 years  [ ] 7-10 years  Other; please specify  **2.8a: How many weeks/months pregnant are you now?**  [ ] 1^st^ month of pregnancy (1-4 weeks)  [ ] 2nd month of pregnancy (5-8 weeks)  [ ] 3rd month of pregnancy (9-13 weeks)  [ ] 4th month of pregnancy (14-17 weeks)  [ ] 5th month of pregnancy (18-21 weeks)  [ ] 6th month of pregnancy (22-26 weeks)  [ ] 7th month of pregnancy (27-30 weeks)  [ ] 8th month of pregnancy (31-35 weeks)  [ ] 9th month of pregnancy (36-40 weeks)  [ ] Currently not pregnant, but experienced MdDS during pregnancy |  |
| **2.9a: What are/were your symptoms like during pregnancy compared to before pregnancy?**  [ ] Significantly better  [ ] Slightly better  [ ] Significantly worse  [ ] Slightly worse  [ ] No change |  |
| **2.10a: Did your MdDS symptoms change throughout your pregnancy/ Are your MdDS symptoms changing throughout your pregnancy?**  [ ]Yes  [ ] No  **2.11a: Select the answer that most accurately describes the nature of your symptoms during pregnancy:**  [ ] Symptoms fluctuate day by day  [ ] Symptoms fluctuate during the day  [ ] Symptoms are stable day by day  [ ] Symptoms fluctuate depending on the stressors  [ ] Symptoms seem to have a cyclical pattern throughout the pregnancy  [ ] Symptoms seem to have a random nature throughout the pregnancy |  |
| **If NO to prior MdDS:**  **2.3b: Who initially diagnosed you with MdDS:**  *Select one answer:*    [ ] Neurologist  [ ] Otolaryngologist  [ ] Physiotherapist  [ ] ENT  [ ] Self-diagnosed  [ ] Other |  |
| **2.4b: Who diagnosed you with MdDS after your initial diagnosis: (e.g. if you were self-diagnosed initially and received an official diagnosis subsequently) and was the specialist/health care professional confident in the diagnosis?**  *Select one answer: (check box will appear next to options listed below for ‘confident in diagnosis’ response)*  [ ] Neurologist  [ ] Otolaryngologist  [ ] Physiotherapist  [ ] ENT  [ ] Other  [ ] N/A as initial diagnosis was the only diagnosis  [ ] N/A as self-diagnosed is the only diagnosis |  |
|  |  |
| **2.5b: To the best of your knowledge, what was the event that induced your MdDS?**  [ ] A motion event (e.g. boat ride, flight, etc.)  [ ] No clear cause  [ ] Other (e.g. surgery, panic attack, etc.)  **2.6b: Please describe what you believe induced your MdDS**  **2.7b: When did your symptoms start?**  [ ] 1^st^ month of pregnancy (1-4 weeks)  [ ] 2nd month of pregnancy (5-8 weeks)  [ ] 3rd month of pregnancy (9-13 weeks)  [ ] 4th month of pregnancy (14-17 weeks)  [ ] 5th month of pregnancy (18-21 weeks)  [ ] 6th month of pregnancy (22-26 weeks)  [ ] 7th month of pregnancy (27-30 weeks)  [ ] 8th month of pregnancy (31-35 weeks)  [ ] 9th month of pregnancy (36-40 weeks)  **2.8b: How many weeks/months pregnant are you now?**  [ ] 1^st^ month of pregnancy (1-4 weeks)  [ ] 2nd month of pregnancy (5-8 weeks)  [ ] 3rd month of pregnancy (9-13 weeks)  [ ] 4th month of pregnancy (14-17 weeks)  [ ] 5th month of pregnancy (18-21 weeks)  [ ] 6th month of pregnancy (22-26 weeks)  [ ] 7th month of pregnancy (27-30 weeks)  [ ] 8th month of pregnancy (31-35 weeks)  [ ] 9th month of pregnancy (36-40 weeks)  [ ] Currently not pregnant, but experienced MdDS during pregnancy |  |
|  |  |
| **2.9b: Did your MdDS symptoms change throughout your pregnancy/ Are your MdDS symptoms changing throughout your pregnancy?**  [ ]Yes  [ ] No |  |
| **2.10b: Select the answer that most accurately describes the nature of your symptoms during pregnancy:**  [ ] Symptoms fluctuate day by day  [ ] Symptoms fluctuate during the day  [ ] Symptoms are stable day by day  [ ] Symptoms fluctuate depending on the stressors  [ ] Symptoms seem to have a cyclical pattern throughout the pregnancy  [ ] Symptoms seem to have a random nature throughout the pregnancy |  |

| **2.12: What was/is your rating of MdDS symptoms on a good day during pregnancy?**  *(0- symptom free, 5- moderate, 10- most severe)*  [0] [1] [2] [3] [4] [5] [6] [7] [8] [9] [10]  **2.13: What was your rating of MdDS symptoms on a good day prior to pregnancy?**  *(0- symptom free, 5- moderate, 10- most severe)*  [0] [1] [2] [3] [4] [5] [6] [7] [8] [9] [10] [N/A – did not have MdDS prior] |
| --- |
| **2.14: What was/is your rating of MdDS symptoms on a bad day during pregnancy?**  *(0- symptom free, 5- moderate, 10- most severe)*  [0] [1] [2] [3] [4] [5] [6] [7] [8] [9] [10]  **2.15: What was your rating of MdDS symptoms on a bad day prior to pregnancy?**  *(0- symptom free, 5- moderate, 10- most severe)*  [0] [1] [2] [3] [4] [5] [6] [7] [8] [9] [10] [N/A – did not have MdDS prior] |
| **2.16: Prior to pregnancy, In which position did you experience motion symptoms the most?**  *Select one answer:*  [ ] Standing  [ ] Sitting  [ ] Lying down  [ ] N/A did not have MdDS prior |
| **2.17: During pregnancy, in which position did/do you experience motion symptoms the most?**  *Select one answer:*  [ ] Standing  [ ] Sitting  [ ] Lying down on your side  [ ] Lying down on your back *(despite this can cause dizziness also in non MdDS patients)*  [ ] Generally lying down  **2.18: During pregnancy, in which position did/do you experience dizziness the most?**  *Select one answer:*  [ ] Standing  [ ] Sitting  [ ] Lying down on your side  [ ] Lying down on your back  [ ] Generally lying down  [ ] N/A I do/did not experience dizziness during pregnancy  **2.19: Prior to pregnancy, which motion symptoms did you experience?**  *Select all that apply:*  [ ] rocking (back/front)  [ ] swaying (side/side)  [ ] bobbing (up/down)  [ ] N/A did not have MdDS prior  **2.20: During pregnancy, which motion symptoms did/do you experience?**  *Select all that apply:*  [ ] rocking (back/front)  [ ] swaying (side/side)  [ ] bobbing (up/down) |
| **2.21: Prior to pregnancy, which motion symptom was the most apparent?**  *Select one answer:*  [ ] rocking (back/front)  [ ] swaying (side/side)  [ ] bobbing (up/down)  [ ] N/A did not have MdDS prior  **2.22: During pregnancy, which motion symptom was/is the most apparent?**  *Select one answer:*  [ ] rocking (back/front)  [ ] swaying (side/side)  [ ] bobbing (up/down) |
| **2.23 During your pregnancy, does/did your mood influence your symptoms?**  *Select one answer:*  [ ] Yes  [ ] No  **2.24: If you are pregnant, when are your symptoms highest during the day, or when you were pregnant, when were your symptoms highest during the day ?**  [ ] As soon as you wake up (6-9am)  [ ] Mid morning (11am)  [ ] Lunch time (12-2pm)  [ ] After lunch (2pm)  [ ] Mid Afternoon (4pm)  [ ] Before dinner (6pm)  [ ] After dinner (8-9pm)  [ ] Before to go to bed (9-11pm)  [ ] High symptom time changes day by day  **2.25: During your pregnancy, what were the symptoms you experienced?**    **2.26: Did you discuss the management of your MdDS symptoms with your gynaecologist/obstetrician?**  [ ]Yes  [ ] No  **2.27: What were the suggestions? And do you believe that they helped?**  **2.28:** **Had your** **gynaecologist/obstetrician heard of MdDS before meeting you?**  [ ]Yes  [ ] No  [ ] Not sure  [ ] I did not discuss MdDS with my gynaecologist/obstetrician  **2.29: Were you on any form of hormonal contraception prior to your pregnancy? I.e. Oral contraceptive pill, nuvaring, hormonal patches, implanon)**  [ ]Yes  [ ] No  **2.30: If yes, for how long:**  Open Answer:  **2.32: Is there anything you would like to add to this section?** |

| **3. TRIGGERS** |
| --- |
| **3.1: What are the triggering factors that would make your symptoms worse during pregnancy?**  [ ] after a car ride  [ ] drinking 1-2 drinks of alcohol  [ ] drinking caffeinated beverages  [ ] working on a computer or cell phone  [ ] in a department store  [ ] in a supermarket/grocery store  [ ] watching movies  [ ] weather change  [ ] stress  [ ] lack of sleep  [ ] dehydration  [ ] hunger  [ ] certain foods, please specify  [ ] loud noises  [ ] bright lights  [ ] flashing lights  [ ] vibration (e.g. massage chair, hand held massager, floors that vibrate if close to plant room, elevator room, air conditioning unit)  [ ] elevator ride  [ ] escalator ride  [ ] other  **3.2: Is there anything you would like to add about your triggers or any experience that you feel is appropriate to this section?** |
